# The TNF-Alpha Inducing Protein is Associated With Gastric Inflammation and Hyperplasia in a Murine Model of *Helicobacter pylori* Infection

**DOI:** 10.3389/fphar.2022.817237

**Published:** 2022-02-14

**Authors:** Lindsay Morningstar-Wright, Steven J. Czinn, M. Blanca Piazuelo, Aditi Banerjee, Renata Godlewska, Thomas G. Blanchard

**Affiliations:** ^1^ GeneDx, Gaithersburg, MD, United States; ^2^ Department of Pediatrics, University of Maryland School of Medicine, Baltimore, MD, United States; ^3^ Department of Medicine, Vanderbilt University School of Medicine, Nashville, TN, United States; ^4^ Department of Bacterial Genetics, Faculty of Biology, University of Warsaw, Warsaw, Poland

**Keywords:** tipα, *Helicobacter pylori*, gastritis, hyperplasia, inflammation

## Abstract

*Helicobacter pylori* (*H. pylori*) is a Gram-negative bacterium that colonizes the human stomach leading to the development of chronic gastritis, peptic ulcers and gastric adenocarcinoma. A combination of host, environment and bacterial virulence factors contribute to disease development. The *H. pylori* TNFα inducing protein (Tipɑ) is a virulence factor shown to induce multiple pro-inflammatory cytokines in addition to TNFα *in vitro*. The goal of the present study was to elucidate the role of Tipα in promoting inflammation *in vivo* and to identify the molecular pathways associated with Tipα associated virulence. Mice were infected with wild-type Sydney strain (SS1) or a *tipα* mutant (Δ*tipα*) for 1 month and 4 months. We also completed a second 4 months infection including a 1:1 SS1 to Δ*tipα* co-infected group in addition to SS1 and Δ*tipα* infected groups. The expression of TNFα, and KC were significantly higher in the SS1 infected group compared to both uninfected control (naïve) and Δ*tipα* groups. Mice infected with Tipα expressing SS1 induced more severe histological gastritis and developed hyperplasia compared to Δtipα infected mice. Microarray analysis of gastric epithelial cells co-cultured with recombinant Tipα (rTipα) demonstrates up-regulation of the NFκB pathway. This data suggest Tipα plays an important role in *H. pylori* induced inflammation.

## Introduction

***H*** *elicobacter pylori*, a Gram- negative bacterium, colonizes the human stomach in approximately half of the world’s population. All infected individuals develop histologic gastritis, which persists throughout the life of the host unless treated (Kusters et al., 2006). Although greater than 80% of individuals remain asymptomatic, chronic *H. pylori* induced gastritis is a risk factor for the development of gastroduodenal diseases such as gastric adenocarcinoma, peptic ulcer disease and MALT lymphoma (Marshall and Warren, 1984; Parsonnet et al., 1994; Parsonnet and Isaacson, 2004). The mode of transmission of *H. pylori* into the human host is poorly characterized but largely believed to occur person to person through the fecal-oral or oral-oral route. Upon entering the stomach, *H*. *pylori* is able to penetrate the mucus layer to colonize the gastric epithelium. Gastric epithelial cells infected with *H. pylori* secrete a host of different cytokines including TNF-alpha (TNF-α), interleukin-1 (IL-1), interleukin-6 (IL-6) and various chemokines to promote inflammation and influence the immune response (Sharma et al., 1995; Beales, 2002; Ricci et al., 2011; Posselt et al., 2013). *H. pylori* relies on the production of several virulence factors including the CagA pathogenicity island which encodes a type four secretion system (T4SS), VacA and urease to interact with gastric epithelial cells to allow for the promotion of colonization and pathogenesis (Covacci et al., 1993; Blaser et al., 1995; Backert and Blaser, 2016; Junaid et al., 2016). A large body of research has focused on how these bacterial factors interact with the host to facilitate pathogenesis. These factors however do not completely account for the host response that can lead to gastric disease.

The TNF-alpha inducing protein (Tipα) is a small, secreted protein from *H. pylori* (Suganuma et al., 2001; Suganuma et al., 2005)*.* It is an *H. pylori* specific virulence factor with no known homology to other *H. pylori* virulence factors or any other existing proteins. Structural analysis indicates that the functional Tipα protein consists of a homodimer approximately 37 kDa in size (Jang et al., 2009; Gao et al., 2012). Using recombinant Tip-α, studies have shown the protein binds nucleolin on the gastric epithelial cell surface and enters the cytoplasm leading to the production of TNFα and other pro-inflammatory cytokines. This is largely believed to take place through the induction of nuclear factor-κB (NF-κB) (Suganuma et al., 2001; Suganuma et al., 2005; Suganuma et al., 2006). Both TNFα and NF-κB play key roles in inflammation and the development of cancer, potentiating the role of Tipα as a carcinogenic factor that contributes to the development of gastric adenocarcinoma. The goal of the present study was to elucidate the potential role of Tipα in promoting inflammation *in vivo*, and to identify the molecular events through with Tipα mediates the cellular response. We evaluated how the host response differs between mice infected with wild type *H. pylori* SS1 (SS1) and a *Tipα* knockout mutant (Δ*tipα*). We also performed microarray analysis on mouse gastric epithelial cells co-cultured with SS1 or Δ*tipα* to identify differences in the host cellular response. We now report that Tip*α* contributes significantly to the transcriptional activation of pro-inflammatory cytokines and the development of gastritis and hyperplasia.

## Materials and Methods

### Bacterial Strains

*H. pylori* Sydney Strain (SS1) (Lee et al., 1997) was grown on Columbia agar (Difco, Detroit, MI) supplemented with 7% horse blood with the antibiotics trimethoprim (20 μg/ml), vancomycin (6 μg/ml), cefsulodin (16 μg/ml) and amphotericin B (2.5 μg/ml) (antibiotics from Sigma-Aldrich, St. Louis, MO). The *H. pylori tipα* mutant (Δ*tipα*) was grown on Columbia agar plates supplemented with 7% horse blood and 50 μg/ml kanamycin (Corning, Corning, New York). Strains were grown at 37°C in a humidified in incubator with 10% CO_2_. *H. pylori* SS1 growth in liquid culture was performed by harvesting bacteria from Columbia blood agar plates in 1 ml of *Brucella* Broth (Difco, Detroit, MI) and transferred to 10 ml *Brucella* broth supplemented with 10% fetal bovine serum (FBS) (Invitrogen, Carlsbad, CA) and 6 μg/ml vancomycin (Sigma) or 50 μg/ml kanamycin (Corning) in T-25 flasks (Corning, New York). Liquid cultures were maintained at 37°C with 10% CO_2_.

### Generation of *H. pylori* SS1 *Tipα* Mutant

The *tipα* knockout mutant (Δ*tipα*) was generated in Sydney strain (SS1) by allelic replacement following a previously reported protocol (Chalker et al., 2001). Briefly, a kanamycin resistance cassette (*aph3*) from *Campylobacter jejuni* was inserted between two fragments of the *tipα* gene. *H. pylori* SS1 genomic DNA was extracted following the Qiagen Genomic DNA protocol (Qiagen, Valencia, CA). *H. pylori* strain HP0596 (NC_000915.1) was used as a reference sequence to amplify the SS1 *tipα* gene by PCR to generate two separate fragments (R1 and R2). The R1 was flanked using PCR primer design by a XbaI restriction enzyme site at the 5′ end and overlap with the upstream Kanamycin gene cassette at the 3’ end using primers XbaI-FP (5′-CCG​ATA​TCT​AGA​GTG​TTA​GAA​AAA​TCT​TTT​TT-3´) and 5prime*aph3* (5′- TTA​TTA​TTT​CCT​TCC​TCT​TTT​CTA​CAG​TAT​TTA​AAG​ATA​CAT​TTG​GAA​AAA​TAA​GCC​TC-3´). R2 was flanked by overlap with the downstream kanamycin gene cassette and an XbaI restriction enzyme site at the 5′ and 3′ end of the R2 fragment respectively, using primers 3prime*aph3* (5′- GTA​CCT​AGA​TTT​AGA​TGT​CTG​AAT​TCG​TAA​CCA​ACC​GCA​TCA​AGC​AAA​AG-3´) and XbaI-RP (5′- GAT​GCC​TCT​AGA​CTA​CAT​GGC​TAT​AGG​GAC​TT-3´). All PCR products were gel purified using the Qiagen QIAquick Gel Extraction Kit. SS1 was transformed with 1 μg of the resulting product on non- selective Columbia blood agar plates at 37°C. After 24 h, bacteria were harvested and re-plated on selective Columbia blood agar plates supplemented with 50 μg/ml kanamycin and incubated at 37°C. Kanamycin resistant (Kan^r^) colonies were selected 5–7 days post transformation and expanded further on selective Columbia blood agar plates with 50 μg/ml kanamycin.

### Western Blot Analysis

Bacterial cell lysates were collected from wild-type SS1 and Δ*tipα* grown on Columbia blood agar plates. A bacterial cell pellet was resuspended in 10 ml of lysis buffer consisting of 100 mM NaCl, 25 mM Tris base, 10 mM MgCl_2_, 50 μg/ml DNaseI and 0.2 mg/ml lysozyme pH 8.0. The cell suspension was lysed by sonication 4 times with a 50% duty cycle and power setting of 5. The cell lysates were aliquoted and stored at −80°C. For the Western blot, 10 µg of cell lysate was used for SDS-PAGE and blots were developed using Tipα specific antibody, anti-HP0596, obtained from Dr. Elzbieta K. Jagusztyn-Krynicka (Institute of Microbiology, University of Warsaw) at a dilution of 1:1000. For the detection of recombinant Tipα protein (see below), 1 µg of purified protein was used for SDS-PAGE and blots were developed using anti-6xHis antibody from Invitrogen (Carlsbad, CA) at a 1:500 dilution.

### Mouse Infections

C57BL/6 female mice (#000664) were purchased from Jackson Laboratory (Bar Harbor, ME) and used for *H. pylori* infection. Four- to five-week old mice were infected by oral gavage (1 × 10^7^ CFU in .5 ml *Brucella* Broth) with wild-type SS1, Δ*tipα* or a 1:1 ratio of SS1 to Δ*tipα*. Each group (uninfected control, SS1, Δ*tipα* and SS1: Δ*tipα* infected mice) contained 10 animals at each time point with the exception of the Δ*tipα* group at 4 months in the second infection (*n* = 5). Groups of mice were euthanized at either 1 month and 4 months post infection and whole stomachs were harvested. A second 4 months infection included the SS1:Δ*tipα* co-infected group in addition to the SS1 and Δ*tipα* infected group. Mice were housed under pathogen-free conditions in microisolater cages at the University of Maryland Baltimore School of Medicine. This study was carried out in strict accordance with the Guide for the Care and Use of Laboratory Animals of the National Institutes of Health. The protocols were approved by the Institutional Animal Care and Use Committee of the University of Maryland in Baltimore School of Medicine.

### Microbial Load Determination

*Helicobacter pylori* was quantified from total DNA using the DNeasy Blood and Tissue kit from Qiagen (Cat. #69504) with an additional 10 min incubation at 95°C following the initial digestion step. Quantitative PCR (qPCR) was used to amplify DNA with SYBR Green (Thermo Scientific, Cat. # 4309155) using *H. pylori 16SrRNA* primers (Table 1) and comparison to a standard curve consisting of SS1 chromosomal DNA as previously described (He et al., 2002; Liu et al., 2008). Bacterial load was reported as the number of *16SrRNA* copies per Gram of tissue. To quantify Δ*tipα* bacteria in the coinfected mice, kanamycin (*aph3*) primers were used (Table 1).

**TABLE 1:** Oligonucleotide sequences for quantitative PCR (qPCR).

Gene	Forward primer (5′-3′)	Reverse primer (5′-3′)	Annealing temp (°C)
*H. pylori 16SrRNA*	ttt​gtt​aga​gaa​gat​aat​gac​ggt​atc​taa​c	cat​agg​att​tca​cac​ctg​act​gac​tat​c	55
*Aph3*	gag​gct​tta​ttt​ttc​caa​atg​tat​ctt​taa​ata​ctg​tag​aaa​ag	ctt​tgc​ttg​atg​cgg​ttg​gtt​acg​aat​tca​gac​atc​taa​atc	55
*Il-17*	atc​cct​caa​agc​tca​gcg​tgt​c	ggg​tct​tca​ttg​cgg​tgg​aga​g	55
*Tnfα*	tcc​cag​gtt​ctc​ttc​aag​gga	ggt​gag​gag​cac​gta​gtc​gg	55
*Il-10*	ccc​tgg​gtg​aga​agc​tga​ag	cac​tgc​ctt​gct​ctt​att​ttc​aca	55
*Ifn-γ*	cat​ggc​tgt​ttc​tgg​ctg​tta​ctg	gtt​gct​gat​ggc​ctg​att​gtc​ttt	55
*Kc*	caa​tgc​gct​gcg​ctg​tca​gtg	ctt​ggg​gac​acc​ttt​tag​cat​c	55
*Gapdh*	cca​ggt​tgt​ctc​ctg​cga​ctt	cct​gtt​gct​gta​gcc​gta​ttc​a	55
*Il-18*	atg​act​tcc​aag​ctg​gcc​gtg​gct	tct​cag​ccc​tct​tca​aaa​act​tct​c	55
*Il-1β*	aac​ctg​ctg​gtg​tgt​gac​gtt​c	cag​cac​gag​gct​ttt​ttg​ttg​t	55
*Foxp3*	cct​gaa​gtt​cat​ctg​cac​cac​c	ctg​ctg​gta​gtg​gtc​ggc​gag​c	55
*Il-6*	atg​aac​tcc​ttc​tcc​aca​agc​gc	gaa​gag​ccc​tca​ggc​tgg​act​g	55

### Histopathology

A longitudinal strip of the entire length of the stomach from forestomach to duodenum was fixed in 10% buffered formalin and paraffin embedded. H&E stained 5 μm sections were graded in a blinded fashion by a clinical pathologist. The corpus and antrum of the stomach were given separate scores using a 0–3 scale (normal, mild moderate, marked) assessing the presence of acute and chronic inflammation. Acute inflammation was defined as the presence of polymorphonuclear neutrophils, and chronic inflammation as the presence of mononuclear leukocytes, as previously described (Dixon et al., 1996). Antral or corporal inflammation consists of the combined score of chronic and acute inflammation, with a maximum score of six in each anatomic location. Global inflammation was calculated using the total of all inflammation scores (acute and chronic in antrum and corpus), with a maximum score of 12. Sections were also graded for mucosal hyperplasia using a 0–3 scale.

### Quantitative Real-Time PCR for Cytokines

Gene expression analysis was performed as previously described (Shiu et al., 2015). RNA was isolated from longitudinal strips of stomach tissue from each group using the RNeasy kit (Qiagen, Cat #74104) and converted to cDNA using Quantitect reverse transcription kit (Qiagen, Cat #205311). PCR amplification was performed using SYBR Green (Thermo Scientific, Cat. # 4309155) in reaction volumes of 20 µL using 10 µM of primer and 100 ng cDNA. Primers used are listed in Table 1. Relative gene expression changes were calculated using the 2^−ΔΔCT^ method and expression was normalized using the housekeeping gene glyceraldehydes 3-phosphate dehydrogenase (*Gapdh*).

### Mouse Gastric Epithelial Cell Line

GSM06 cells, a mouse gastric epithelial cell line generated from a T antigen transgenic mouse was obtained from RIKEN (Wako, Saitama, Japan). Cell cultures were maintained in Dulbecco’s Modified Eagle Media (DMEM) (Corning) supplemented with 10% FBS (Gemini Bio-Products), 1% v/v Anti-/Anti- (Gemini Bio-Products) and 1% v/v ITS (Insulin, transferrin and sodium selenite) (Thermo Fisher Scientific). Cells were maintained and expanded at 33°C with 5% CO_2_. GSM06 cells were co-culture with recombinant Tipα at 37°C with 5% CO_2_ during experimentation.

### Production of Recombinant Tipα Protein in *Escherichia coli*

A recombinant Tipɑ (rTipɑ) construct was purchased from Addgene (Cambridge, MA) and was created by cloning the *H. pylori tipɑ* sequence into a pET28b expression vector incorporating an N-terminal 6X Histidine tag (Jang et al., 2009). The recombinant plasmid was transformed into *E. coli* BL21 (DE3) competent cells (New England Biosystems, Ipswich, MA) and plated on LB agar (Difco) plates supplemented with 30 μg/ml kanamycin. We used colony PCR amplification of the *tipɑ* gene sequence to select colonies that were positive for the construct (forward primer 5′- CAG​GTT​GGA​TCC​GTG​TTA​GAA​AAA​TCT​TTT​TT-3′ and reverse primer 5′- TTC​AGG​GAT​ATC​GGT​ACA​TCC​CTA​GGT​TCG​CG - 3´). Protein expression was carried out in transformed *E. coli* BL21 (DE3) cells following the suggested protocol by the manufacturer (New England Biosystems). A 25 ml starter culture was used to inoculate 500 ml of LB broth supplemented with 30 μg/ml kanamycin. Recombinant protein expression was stimulated with 0.5 mM Isopropyl β-D-1-thiogalactopyranoside (IPTG) (Sigma-Aldrich) when log phase was reached (OD_600_ = 0.45). Cultures were shaken at 210 rpm overnight at 30°C to maximize protein production. *E. coli* cell pellets were resuspended in a lysis buffer containing 50 mM Tris-HCl, pH7.9, 200 mM NaCl, 10% v/v glycerol and 50 mM imidazole (Jang et al., 2009). Cells were sonicated 4 times in 10-s bursts with 30 s resting period in between bursts. Cell debris was removed by centrifugation and the supernatant was applied to HisPur Ni-NTA Resin (Thermo Fisher Scientific) to purify the recombinant Tipɑ protein following the manufacturer protocol. Purified recombinant Tipɑ was quantified using the Pierce BCA assay (ThermoFisher Scientific) and detected by western blot analysis as described above.

### Recombinant Tipɑ and Epithelial Cell Co-Culture

GSM06, were stimulated for 2 h with 10 μg/ml recombinant Tipɑ (rTipɑ), 100 μg/ml Polymixin B (PMB), or a both rTipɑ and PMB. After 2 h, RNA was isolated using the RNeasy kit (Qiagen, Cat #74104). Total RNA was quantified using the NanoDrop 2000 spectrophotometer and stored at -80°C. Prior to the co-culture, the rTipɑ protein and the *E. coli* LPS control were treated with 100 μg/ml Polymixin B (PMB) for 30 min. The addition of the Polymixin B to the rTipɑ was to eliminate any LPS contamination that could interfere with downstream gene expression analysis (Duff and Atkins, 1982).

### Mouse Microarray

Microarray analysis was completed by the Biopolymer-Genomics Core facility at the University of Maryland School of Medicine. Total RNA from unstimulated (*n* = 2), Polymixin B (PMB) (*n* = 2), rTipɑ treated (*n* = 2) and PMB + rTipɑ treated (n = 2) GSM06 cells were converted to cDNA and hybridized to the Mouse Clariom S array (Applied Biosystems, Waltham, MA) containing >221,300 gene targeting probes. Differential gene expression analysis was performed using Oligo package for R statistical software (Carvalho and Irizarry, 2010). A gene was considered differentially expressed if the *p*-value was <0.05 and the log2-fold change was ≥2 or ≤ -2. The Ingenuity Pathway Analysis (IPA) was completed using the differential gene expression analysis from Oligo to identify entire gene networks that may be attributed to Tipɑ (Kramer et al., 2014). The gene expression and IPA pathway analysis were completed by Dr. Yang Song (The Institute for Genome Sciences, University of Maryland Baltimore).

### Statistical Analysis

Data are presented as mean with standard error of the mean (SEM) calculated by one-way analysis of variance (ANOVA using Graph Pad Prism for Macintosh 5.0c (Graph Pad Software Inc., San Diego, CA) comparing the naïve (uninfected controls), SS1and Δ*tipα* infected mice. Significance between groups was further analyzed using the post hoc Tukey test. *p* values were considered significant if less than 0.05 and are indicated using asterisks * = *p* < 0.05, ** = *p* < 0.01, *** = *p* < 0.001. Each mean and S.E is representative of 10 animals unless otherwise noted.

## Results

**FIGURE 1:**
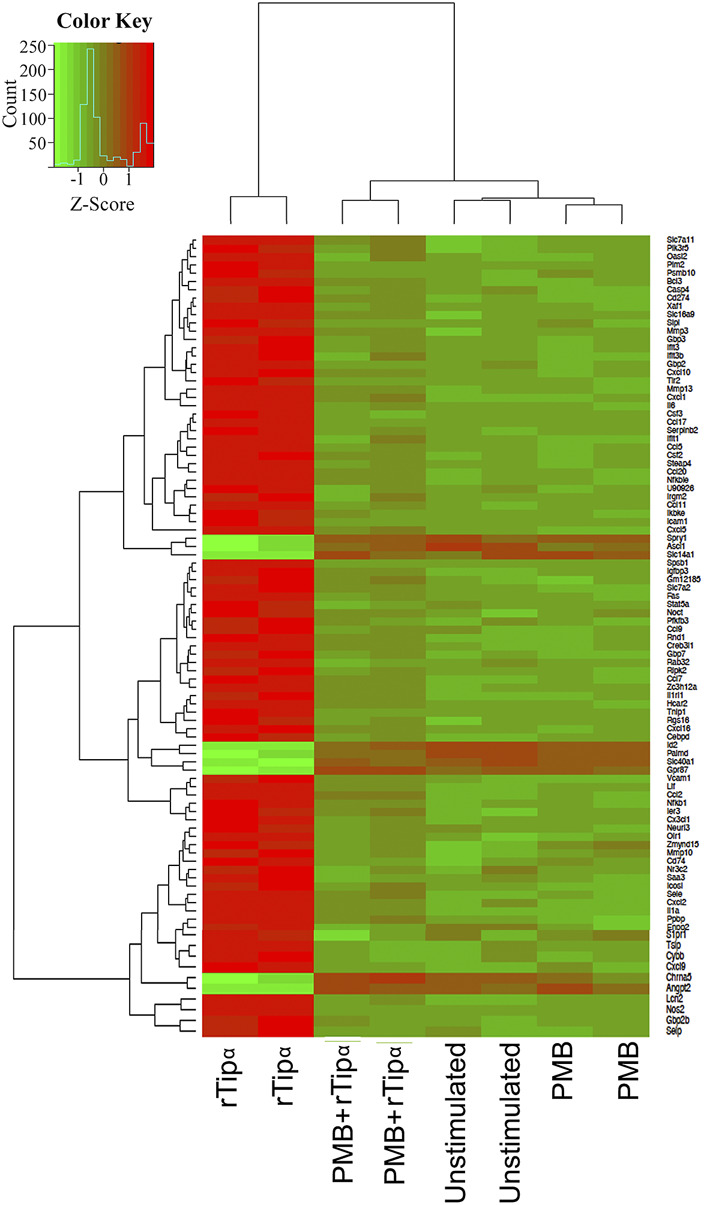
Gene expression across all microarray samples. Heatmap showing differentially expressed genes. Z-scores were filtered with an absolute value ≥1 and *p*-values < .05.

A mouse gastric epithelial cell line, GSM06, was stimulated with recombinant Tipα (rTipα) to investigate how Tipα affects epithelial cells and potentially promotes inflammation. A plasmid construct previously used for Tipα biochemical structural analysis (Addgene) was used to generate recombinant protein (Jang et al., 2009). Post purification, two distinct bands corresponding to the functional rTipα homodimer (39 kDa) and monomer (19 kDa) were evident by immunoblot using an anti- 6X histidine antibody (Supplementary Figure S1) (Suganuma et al., 2005; Kuzuhara et al., 2007a). Recombinant Tipα was pre-treated with Polymixin B (PMB) for 30 min prior to co-culture to neutralize potential LPS contamination. RNA from unstimulated, PMB, rTipα, and rTipα+PMB treated GSM06 cells were used for microarray analysis after 2 h of culture. The 2 h time point for early gene expression events was selected based on separate studies we performed using whole bacteria and the human N87 gastric epithelial cell line. Two comparisons yielded significant gene expression results. Compared to unstimulated cells, rTipα upregulated 107 genes and downregulated nine genes. When rTipα+PMB treatment was compared to rTipα treatment nine genes were found to be upregulated and 85 genes were downregulated. When examining expression across all samples, the rTipα treated cells had a different gene expression pattern compared to the other samples, explaining why comparisons containing rTipα expression data showed significance (Figure 1). A notable number of these genes were chemokines and proinflamamtory genes such as *IL-1*, *Ccl2*, *IL-6* and *Cxcl10*. However, most of the genes that were up-regulated by rTipα failed to be induced in the rTipα+PMB, unstimulated and PMB treated groups.

**FIGURE 2:**
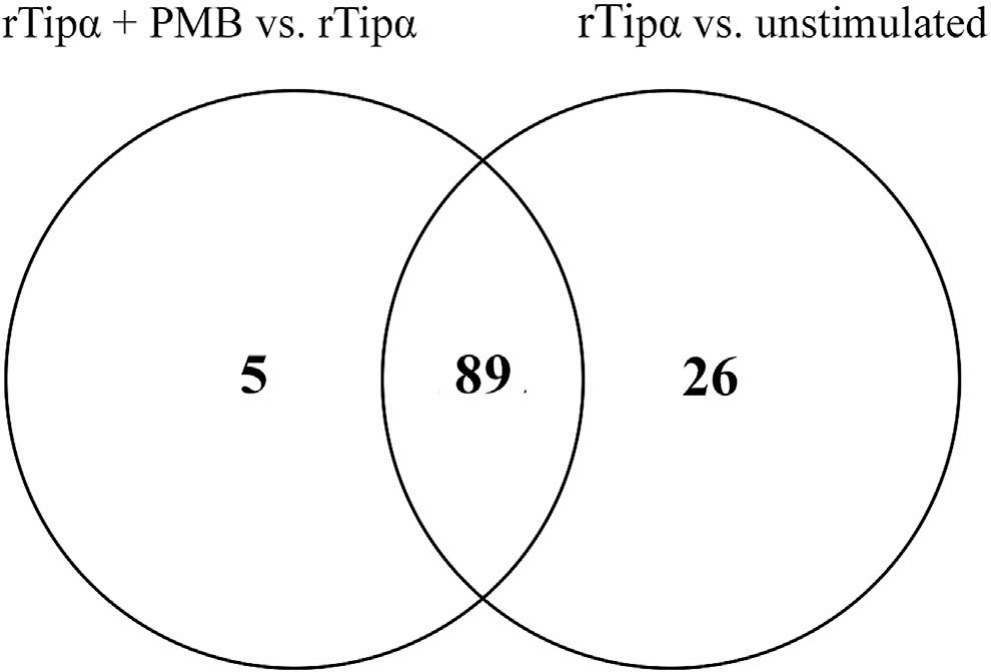
Overlap of genes differentially expressed in both comparisons. This venn digram uses the significant differentially expressed genes from both comparisons to identify unique genes in each dataset. 89 genes are predicted to be influenced by LPS leaving 26 unique genes from rTipα versus unstimulated and five unique genes from rTipα+ PMB versus rTipα.

Differentially expressed genes from the two comparisons described above were compared for overlap between samples to determine if any genes were attributable specifically to Tipα. (Figure 2). Because each comparison contains rTipα without PMB treatment, the 89 gene overlap represents those genes targeted by LPS, leaving 26 genes potentially influenced by Tipα (Table 2). Among the 26 genes are *Nfκb* and *Tnf*, which are predicted to be up regulated in previous Tipα studies (Suganuma et al., 2001; Cheng et al., 2008; Suganuma et al., 2008). IPA analysis yielded results for upstream regulators and canonical pathways that could be attributed to Tipα (Tables 3, 4). In the upstream regulator analysis, common pro-inflammatory mediators were predicted to be activated in addition to TNF (Table 4).

**TABLE 2:** List of 26 unique genes in the rTipα versus unstimulated that could be Tipα gene targets.

Gene name	Description	Fold change
Up-regulated		
*Gm1045*	Predicted gene 1045	3.52
*Gch1*	GTP cyclohydrolase 1	3.37
*Nlrp3*	NLR family, pyrin domain containing 3	2.81
*Nfkbiz*	Nuclear factor of kappa light polypeptide gene enhancer in B cells inhibitor, zeta	2.50
*Nfkbia*	nuclear factor of kappa light polypeptide gene enhancer in B cells inhibitor, alpha	2.49
*Tnip3*	TNFAIP3 interacting protein 3	2.46
*Nr4a3*	Nuclear receptor subfamily 4, group A, member 3	2.43
*Glis3*	GLIS family zinc finger 3	2.42
*Dtx3l*	Deltex 3-like (*Drosophila*)	2.38
*Tnfsf15*	Tumor necrosis factor (ligand) superfamily, member 15	2.36
*Hk2*	Hexokinase 2	2.33
*Tnfaip3*	Tumor necrosis factor, alpha-induced protein 3	2.24
*Wnt7b*	Wingless-type MMTV integration site family, member 7B	2.21
*Nfkbib*	Nuclear factor of kappa light polypeptide gene enhancer in B cells inhibitor, beta	2.18
*Tnf*	Tumor necrosis factor	2.18
*Serpina3n*	Serine (or cysteine) peptidase inhibitor, clade A, member 3N	2.17
*Rhbdf2*	rhomboid 5 homolog 2 (*Drosophila*)	2.12
*Gdpd5*	Glycerophosphodiester phosphodiesterase domain containing 5	2.10
*Hivep2*	Human immunodeficiency virus type I enhancer binding protein 2	2.10
*Ampd3*	Adenosine monophosphate deaminase 3	2.04
*Plscr1*	Phospholipid scramblase 1	2.03
*Cish*	Cytokine inducible SH2-containing protein	2.02
*Fgf7*	Fibroblast growth factor 7	2.01
*Pdgfb*	Platelet derived growth factor, B polypeptide	2.00
Down-regulated		
*Rapgef4*	Rap guanine nucleotide exchange factor (GEF) 4	-2.06
*Sdpr*	Serum deprivation response	-2.00

**TABLE 3:** Upstream regulators predicted to be activated by Tipα. IPA analysis of 26 significant genes after accounting for LPS contamination.

Upstream regulators			
Regulator	Molecule type	Predicted activation state	Activation z-score
IL1A	cytokine	Activated	2.395
IL1B	cytokine	Activated	2.019
TNF	cytokine	Activated	1.172
IL-6	cytokine	Activated	2.372
IFNγ	cytokine	Activated	1.953

**TABLE 4:** Predicted canonical pathways altered by Tipα. IPA analysis of 26 significant genes after accounting for LPS contamination.

Canonical pathways		
Pathway	z-score	Molecules
Acute phase Response Signaling	1	NFKBIA, SERPINA3, NFKBIB, TNF
NF-κB Signaling	-1	NFKBIA, TNFAIP3, NFKBIB, TNF
PPAR Signaling	-2	NFKBIA, NFKBIB, TNF, PDGFB

**FIGURE 3:**
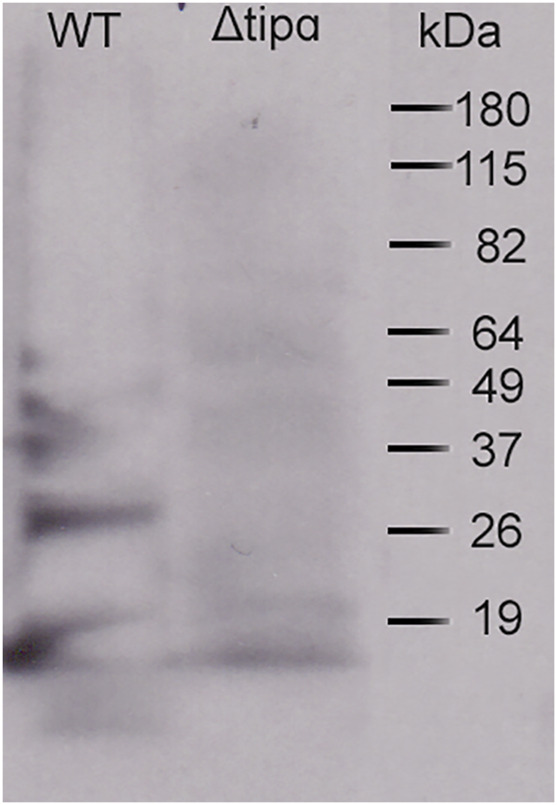
*Helicobacter pylori* ΔtipαSS1 lacks Tipα protein expression. Rabbit anti-Tipα antiserum staining of lysate antigen from WT SS1 and from a ΔtipαSS1 deletion mutant for detection of Tipɑ in a western blot to confirm lack of expression by the mutant.

Most studies to date have focused on *in vitro* effects of Tipɑ using a recombinant protein (rTipɑ) on gastric epithelial cell lines. An *in vivo* study examined mice infected with wild- type Sydney strain (SS1) or a *tipα* mutant for 3 weeks (Godlewska et al., 2008). Both wild- type (WT) and mutant infected mice were able to colonize mice with the mutant showing reduced colonization in comparison to the SS1. We wanted to expand this model of *in vivo* analysis to explore the effects of Tipɑ on the host immune response by comparing mice infected with SS1 and mice infected with a *tipα* mutant (Δ*tipα*). Strain SS1 was used to remain consistent with the previous report (Godlewska et al., 2008), and because SS1 lacks a functional T4SS. The binding of the TFSS to the host epithelial cells induces proinflammation signaling including IL-8 production and downstream events (Fischer et al., 2001). Therefore, the presence of a TFSS would have the potential to mask any inflammatory changes that might result from knocking out Tipα. We first generated a SS1 strain lacking Tipɑ expression confirmed by western blot (Figure 3) and then compared groups of mice infected at 1 month and 4 months. Infection with SS1 at 1 month is typically easy to verify and *H. pylori*-induced inflammation is often apparent. Stomachs from mice collected at 4 months were also included to determine if elements of pathogenesis associated with longer term infection might develop and to compare pathology induced by chronic infection with a shorter term infection.

**FIGURE 4:**
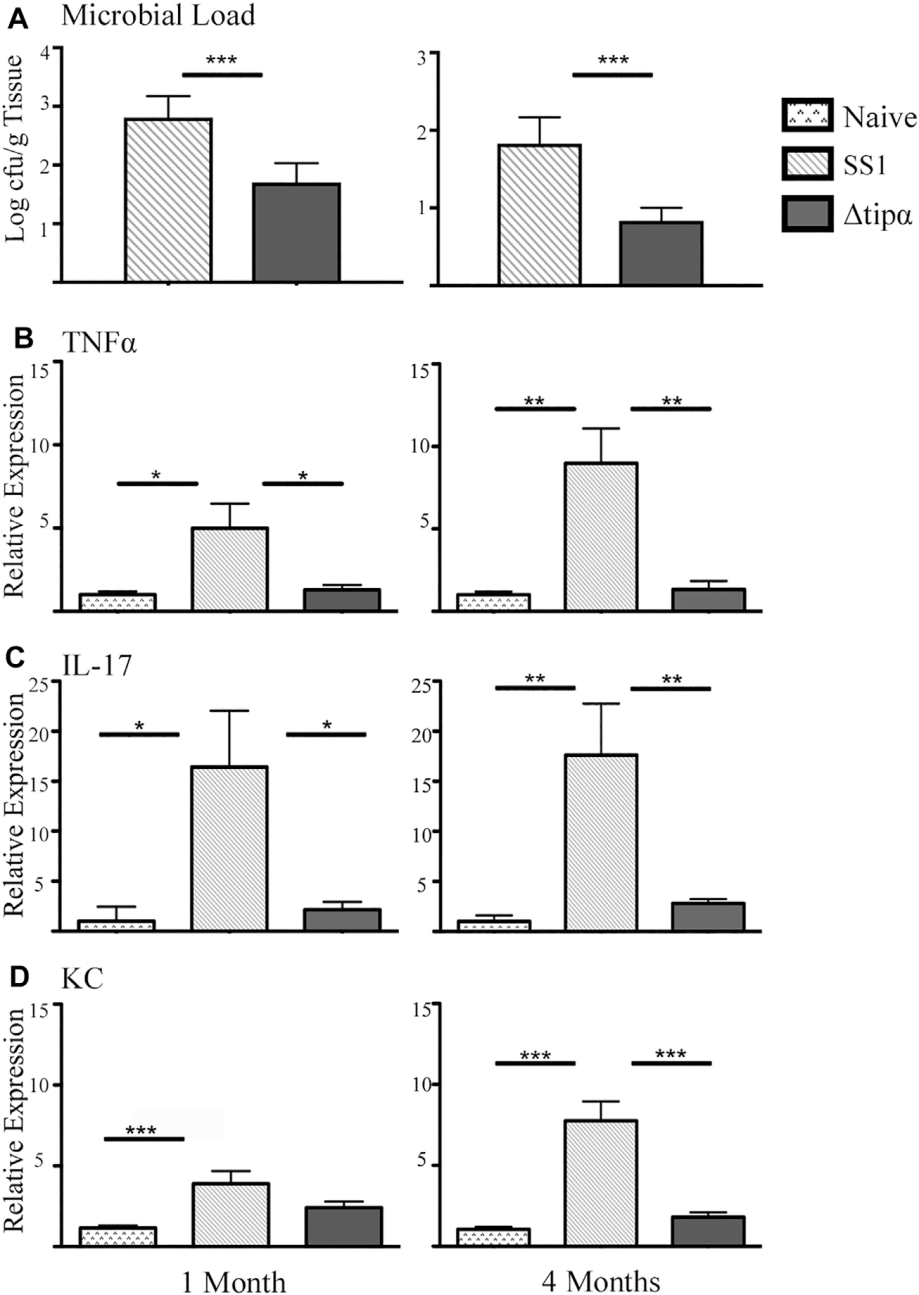
Quantitative PCR (qPCR) microbial load and cytokine expression analysis of SS1 and Δ*tipα* infected mice at 1 month and 4 months post infection (p.i.). **(A)** Microbial load determination by qPCR of *H. pylori 16SrRNA* gene copy number per Gram of tissue shown as mean ± SEM at 1 month and 4 months p.i. (*n* = 10) **(B)** Mean expression of Tnfα in the naïve, SS1 and Δ*tipα* infected mice at 1 month and 4 months p.i. (*n* ≥ 9) using qPCR. **(C)** Mean expression ±SEM of IL-17 in the naïve, SS1 and Δ*tipα* infected mice at 1 month and 4 months p.i. (*n* ≥ 7). **(D)** Mean KC expression ±SEM in the naïve, SS1 and Δ*tipα* infected mice at 1 month and 4 months p.i. (*n* ≥ 7). *p < .05, **p < .01, ***p < .001.

Microbial loads of each group showed infection with *Helicobacter pylori* SS1 (Figure 4A) with an overall reduction of microbial load in the Δ*tipα* infected mice in comparison to WT SS1. We evaluated the transcriptional activition of multiple cytokines commonly found to be present during *H. pylori* infection or in other models of inflammation to examine the role of Tipα in the immune response to *H. pylori* infection. Those demonstrated the significant transcriptional upregulation are shown in Figure 2. TNFα was significantly higher in SS1 infected mice compared to Δ*tipα* infected mice and naïve controls at 1 month (*p* < .05) and this difference increased by 4 months (Figure 4B).

**FIGURE 5:**
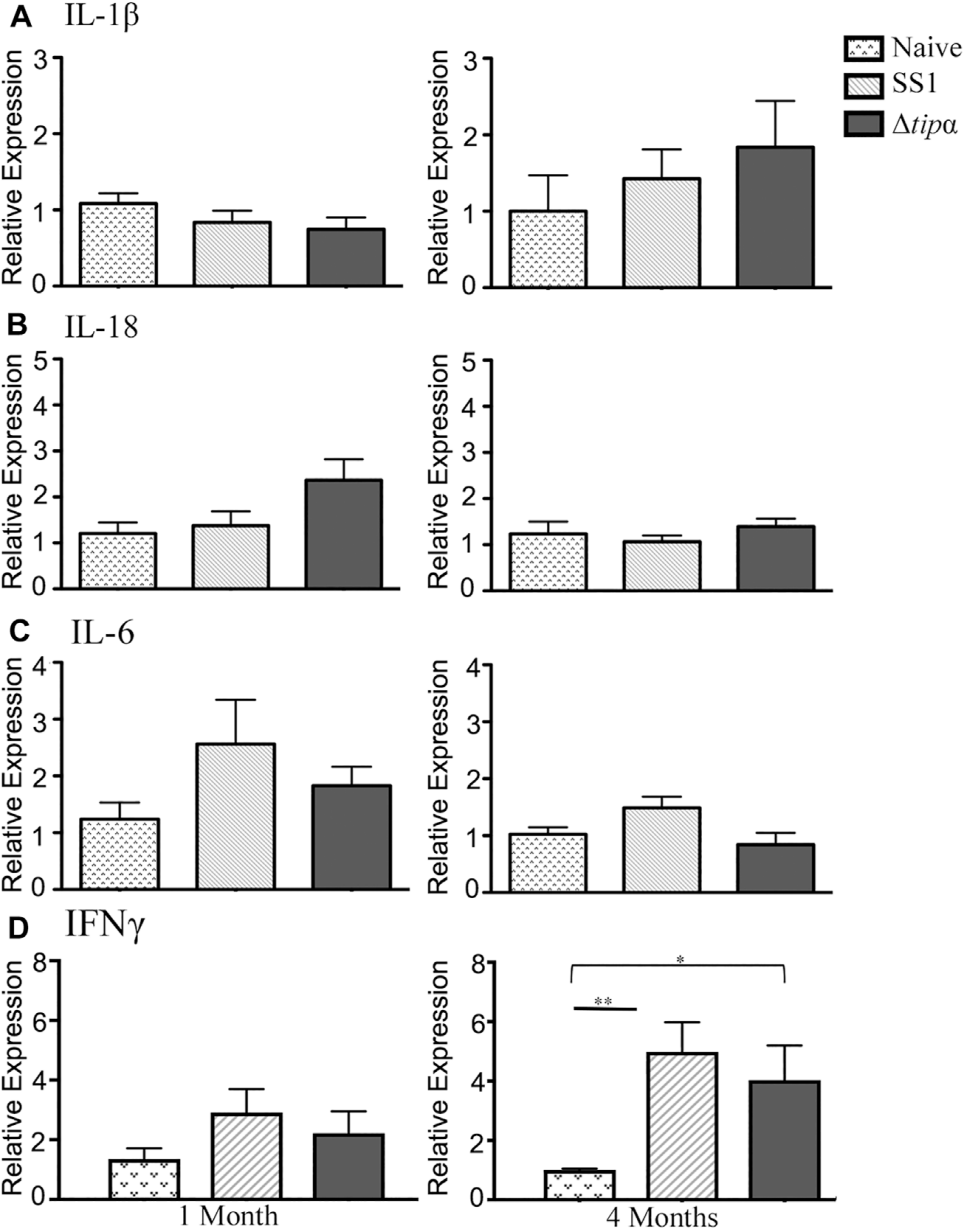
Quantitative PCR (qPCR) expression analysis of SS1 and Δ*tipα* infected mice at 1 month and 4 months post infection (p.i.). **(A)** Mean expression of IL-1β in the naïve, SS1 and Δ*tipα* infected mice at 1 month and 4 months p.i. (n ≥ 9) using qPCR. **(B)** Mean expression ±SEM of IL-18 in the naïve, SS1 and Δ*tipα* infected mice at 1 month and 4 months p.i. (n ≥ 7). **(C)** Mean expression ±SEM of mIL-6 in the naïve, SS1 and Δ*tipα* infected mice at 1 month and 4 months p.i. (n ≥ 7). **(D)** Mean expression ±SEM of IFNγ in the naïve, SS1 and Δ*tipα* infected mice at 1 month and 4 months p.i. (n ≥ 8).

Increased IL-17 expression has been reported in the *H. pylori* infected gastric mucosa (Luzza et al., 2000; Caruso et al., 2008). This cytokine is expressed from a subset of T-helper 17 (Th17) cells and promotes inflammation during *H. pylori* infection. In our experiment IL-17 levels were 6–7 fold higher in the SS1 infected group compared to both the naïve (*p* < .05) and the Δ*tipα* (*p* < .01) infected group at both 1 and 4 months post infection (Figure 4C). KC expression was significantly higher in the SS1 infected group compared to the Δ*tipα* but this difference did not manifest until 4 months (Figure 4D). Cytokines including IL-1β, IL-18, IL-6, and IFNγ were also evaluated but no significant differences were detected between infected groups with the exception of IFNγ mRNA levels which were elevated in both *H. pylori* infected groups in comparison to the naïve, uninfected control (*p* < .05) at 4 months post infection (Figure 5). IL-10 and FoxP3 expression also showed no significant differences between groups (data not shown). Gastric tissues were evaluated post infection to assess specific tissue responses and the overall degree of inflammation.

**FIGURE 6:**
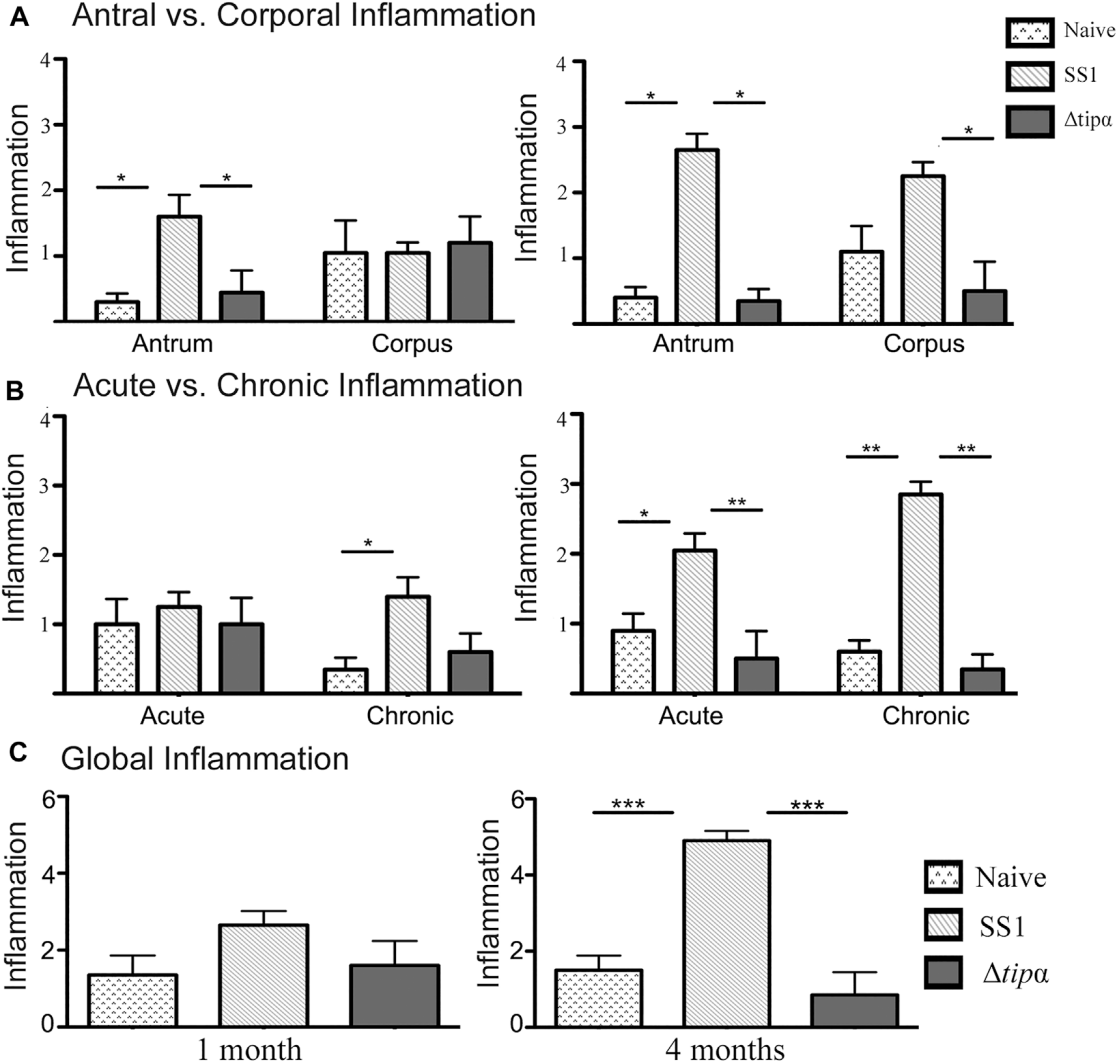
Regional inflammation between SS1 and Δ*tipα* infected mice at 1 month and 4 months post infection. **(A)** Inflammation scores comparing sections of the antrum and corpus of the stomach at 1 month and 4 months post infection. Scores include both acute and chronic inflammation scores, maximum score of 6. **(B)** Inflammation scores comparing the presence of acute and chronic inflammatory markers at 1 month and 4 months post infection. Scores include both antral and corporal inflammation scores, maximum score of 6. **(C)** Total score of inflammation across the whole stomach encompassing antrum and corpus/acute and chronic inflammation scores. Maximum score of 12. *p < .05, **p < .01, ***p <.001.

We assessed inflammation, a measure of the presence of immune cells along the whole stomach including the corpus and antrum. Acute *H. pylori* infection can present with antral predominant gastritis and over time chronic infection can lead to pangastritis or the global presence of inflammation throughout the stomach. At 1 month post infection, higher antral inflammation was present in SS1 infected mice (*p* < .05) which corresponds to the initial colonization of the gastric antrum in *H. pylori* infection (Figure 6A). Antral inflammation increased, as well as the presence of inflammation in the corpus of the stomach in the SS1 infected group when compared to the Δ*tipα* and naïve mice by 4 months (*p* < .05) (Figure 6A). The presence of acute and chronic inflammatory markers was determined by grading histological sections for the presence and degree of neutrophil and mononuclear leukocyte infiltration. Active gastritis is commonly seen in *H. pylori* infection where both neutrophils and mononuclear cells are present within the gastric mucosa (Supplementary Figures S2, S3). By 4 months post infection, the SS1 infected group had a greater presence of acute and chronic inflammatory markers compared to Δ*tipα* infection (*p* < .01) and naïve mice (*p* < .05) (Figure 6B). When inflammation was assessed using a global score (see methods) a significant difference was seen by 4 months post infection in the SS1 infected group compared to the naïve control and Δ*tipα* infected mice (*p* < .001) (Figure 6C).

**FIGURE 7:**
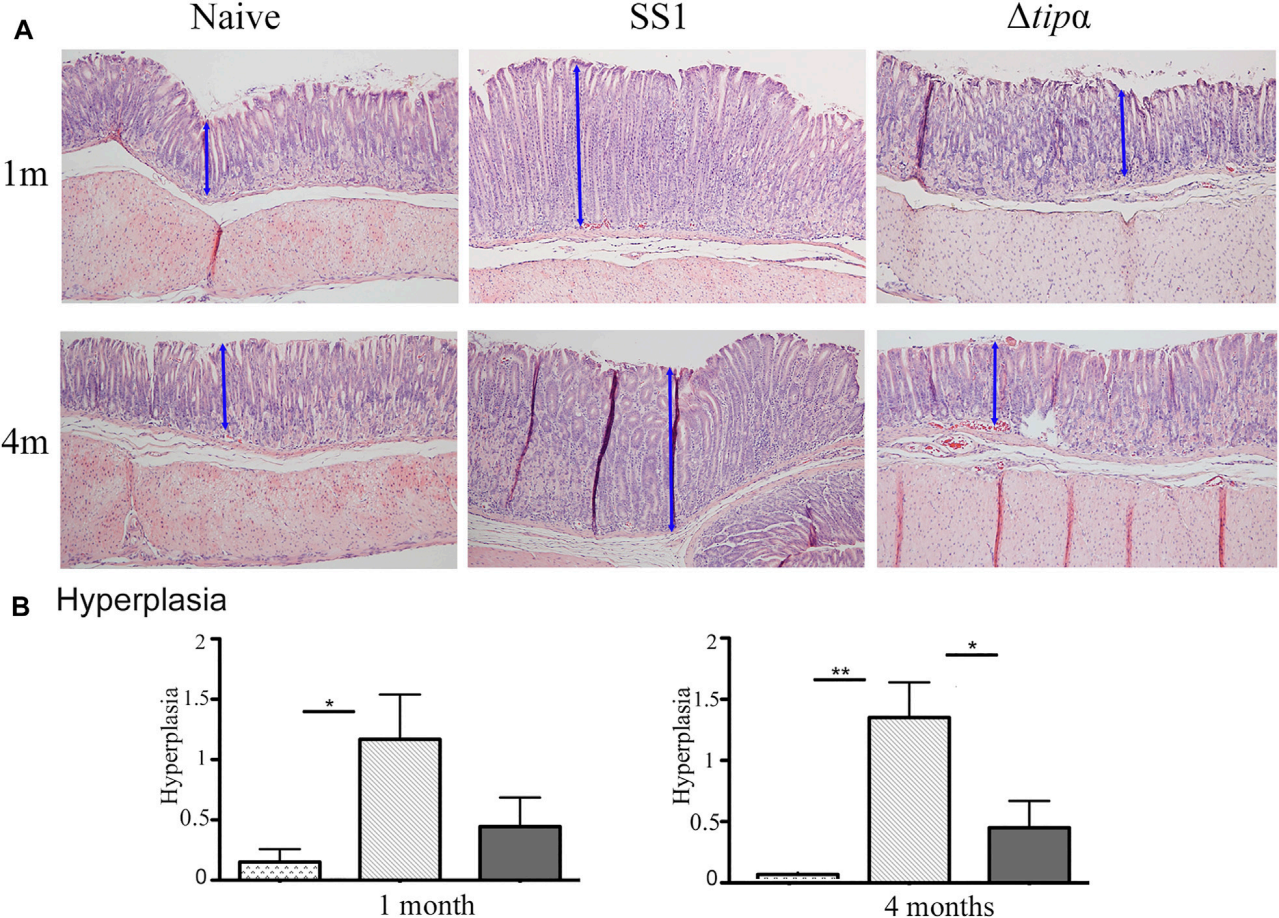
Total inflammation analysis of SS1 and Δ*tipα* infected mice at 1 month and 4 months post infection. **(A)** Representative H&E-stained stomach sections of each group encompassing the transitional zone between the corpus and antrum of the stomach at ×100 magnification. **(B)** Histology scores comparing the presence of hyperplasia between each infected mouse group and naïve, uninfected mice. Maximum score of 3. *p < .05, **p < .01, ***p <.001.

**T**he presence of hyperplasia was examined in gastric tissue samples between the *H. pylori* infected groups and the naïve control. The SS1 infected group showed signs of hyperplasia at 1 month and 4 months whereas little to no hyperplasia was seen in the naïve and Δ*tipα* infected mice (Figure 7). Although hyperplasia is generally associated with chronic infection we observed thickened mucosa and elongated glands by 1 month (indicated by arrows). The presence of hyperplasia indicates the presence of cellular changes that can lead to the loss of glands and atrophic gastritis, predisposing the tissue to cancer. This data, in conjunction with the increased pro-inflammatory cytokine mRNA production and increased presence of histological gastritis in the wild-type infected mice, suggests that Tipɑ contributes to promoting *H. pylori* induced pathology.

**FIGURE 8:**
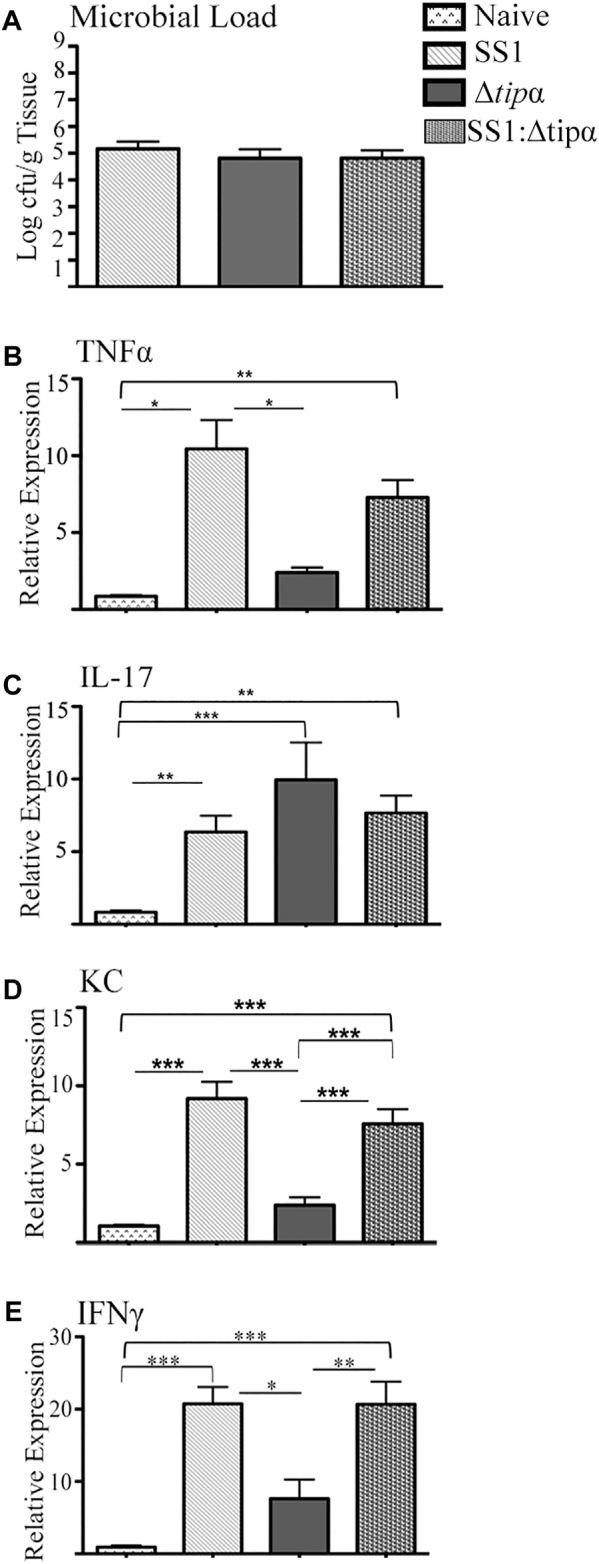
Microbial load and cytokine expression analysis of SS1, Δ*tipα* and SS1:Δ*tipα* infected mice at 4 months post infection. **(A)** Microbial load determination by qPCR of *H. pylori 16SrRNA* gene copy number per Gram of tissue shown as mean ± SEM at 4 months p.i. **(B)** Mean expression of TNFα in the naïve, SS1, Δ*tipα* and SS1:Δ*tipα* infected mice at 4 months p.i. (n ≥ 4) using qPCR. **(C)** Mean expression ±SEM of IL-17 in the naïve, SS1, Δ*tipα* and SS1:Δ*tipα* infected mice at 4 months p.i. (n ≥ 5). **(D)** Mean KC expression ±SEM in the naïve, SS1, Δ*tipα* and SS1:Δ*tipα* infected mice at 4 months p.i (n > 4). **(E)** Mean IFNγ expression ±sem in the naïve, SS1, Δ*tipα* and SS1:Δ*tipα* infected mice at 4 months p.i. (n > 5). *p < .05, **p < .01, ***p <.001.

A second 4 months infection model was performed which included a group co-infected 1:1 with WT SS1 and the Δ*tipα* mutant to determine if the presence of Tipα provides a competitive advantage to the bacteria during infection and to evaluate the host response. All groups had similar levels of bacterial colonization as determined by PCR analysis for *H. pylori* 16S DNA (Figure 8A). Those cytokines demonstrated to increase significantly in transcriptional upregulation in response to infection in Figures 4, 5 were evaluated. TNFα expression was similar to our initial experiment with SS1 infected mice expressing significantly higher levels than Δtipα infected mice (*p* < .05; Figure 8B). Elevation of mRNA levels of this cytokine in the coinfected group of mice more closely resembled SS1 infected mice with expression compared to naïve mice reaching similar differences (*p* < .01). IL-17 mRNA expression levels between Δtipα and SS1 infected mice were not statistically significant (Figure 8C). Expression in the co-infected group was similar to the SS1 and Δ*tipα* mutant infected groups. KC expression however was similar to our initial experiment with SS1 infected mice expressing significantly higher levels than Δtipα infected mice (*p* < .001; Figure 8D). IFNγ levels were higher overall in comparison to the first experiment with SS1 infected mice expressing approximately three fold higher levels than the Δtipα infected group. Similar to TNFα expression, IFNγ levels in the co-infected group were comparable to that of SS1 infected mice (Figure 8E). Overall, the cytokine mRNA host response in co-infected mice therefore resembled that of SS1 infected mice indicating that the presence of Tipα in the WT bacteria was sufficient to dominate the response, even in the presence of bacteria lacking Tipα.

**FIGURE 9:**
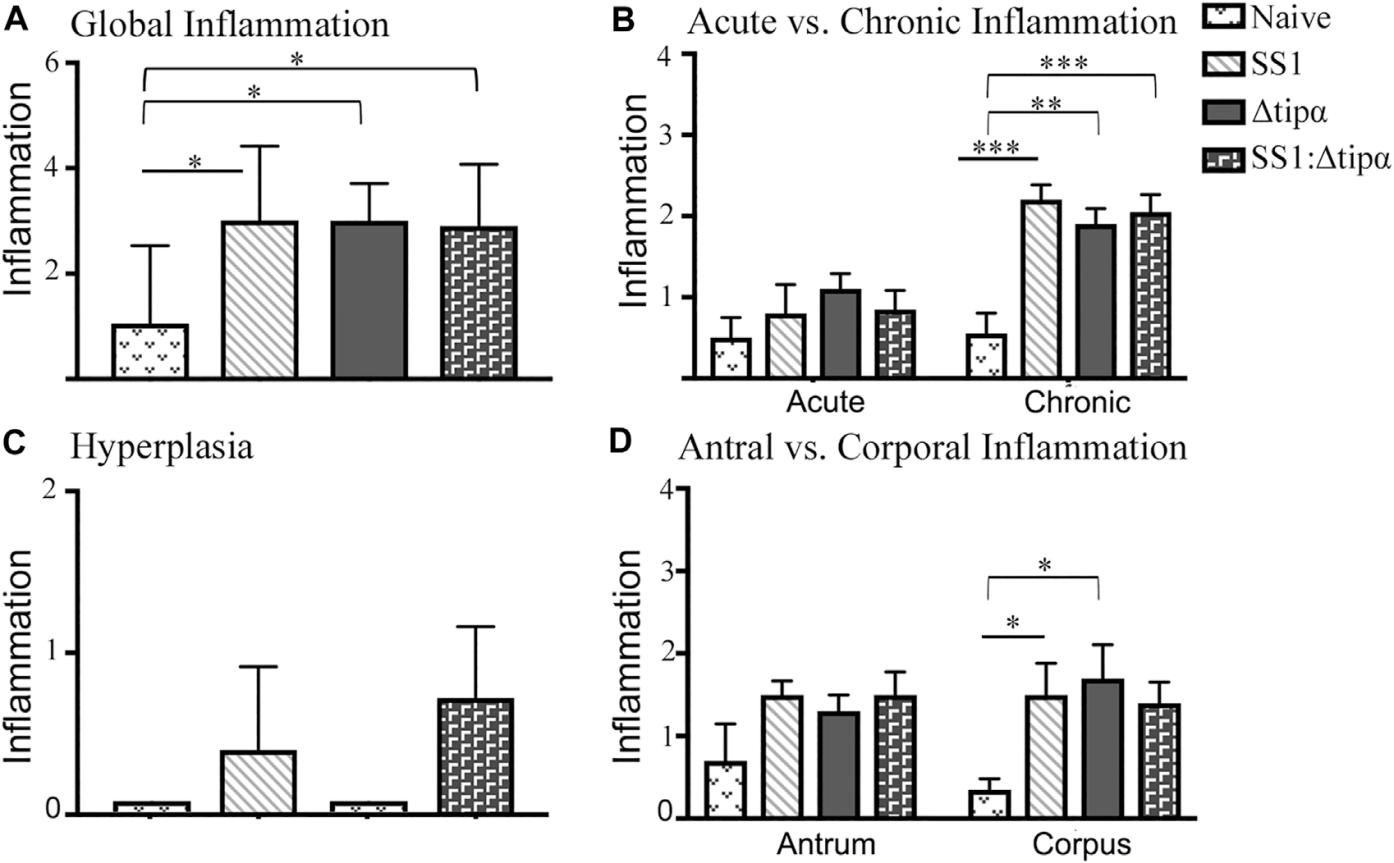
Histology of naïve and SS1-, Δ*tipα-* and SS1:Δ*tipα*-infected mice at 4 months post infection. **(A)** Global inflammation, (sum of antral and corporal acute and chronic inflammation) scores, with a maximum value of 12. **(B)** Acute and chronic inflammation scores at 4 months infection. Scores include both antral and corporal inflammation scores, maximum score of 6. **(C)** Histology scores comparing the presence of hyperplasia between each infected mouse group and naïve, uninfected mice. Maximum score of 3. **(D)** Inflammation scores comparing sections of the antrum and corpus of the stomach at 4 months post infection. Scores include both acute and chronic inflammation scores, maximum score of 6. *p < .05, **p < .01, ***p <.001.

Histologic evaluation of the gastric mucosa in these mice failed to identify meaningful differences between groups with respect to inflammation. All three infected groups had comparable global inflammation scores (Figure 9A). Additionally, all three groups developed chronic inflammation at 4 months that were not quantitatively distinct from each other although the increases compared to naïve control mice varied somewhat (*p* < .01 to *p* < .001; Figure 9B). The inflammation was found to be predominantly in the corpus. Although all three infected groups were statistically similar, only the SS1 and Δ*tipα* mutant infected groups were significantly greater than naïve control mice (Figure 9D). Hyperplasia did not reach the same levels as our previous experiment shown in Figure 7. Only the WT SS1 and the SS1: Δ*tipα* co-infected groups however developed any hyperplasia (Figure 9C), an observation consistent with hyperplasia observed predominantly in the SS1 infected group in the experiment shown in Figure 7.

**FIGURE 10:**
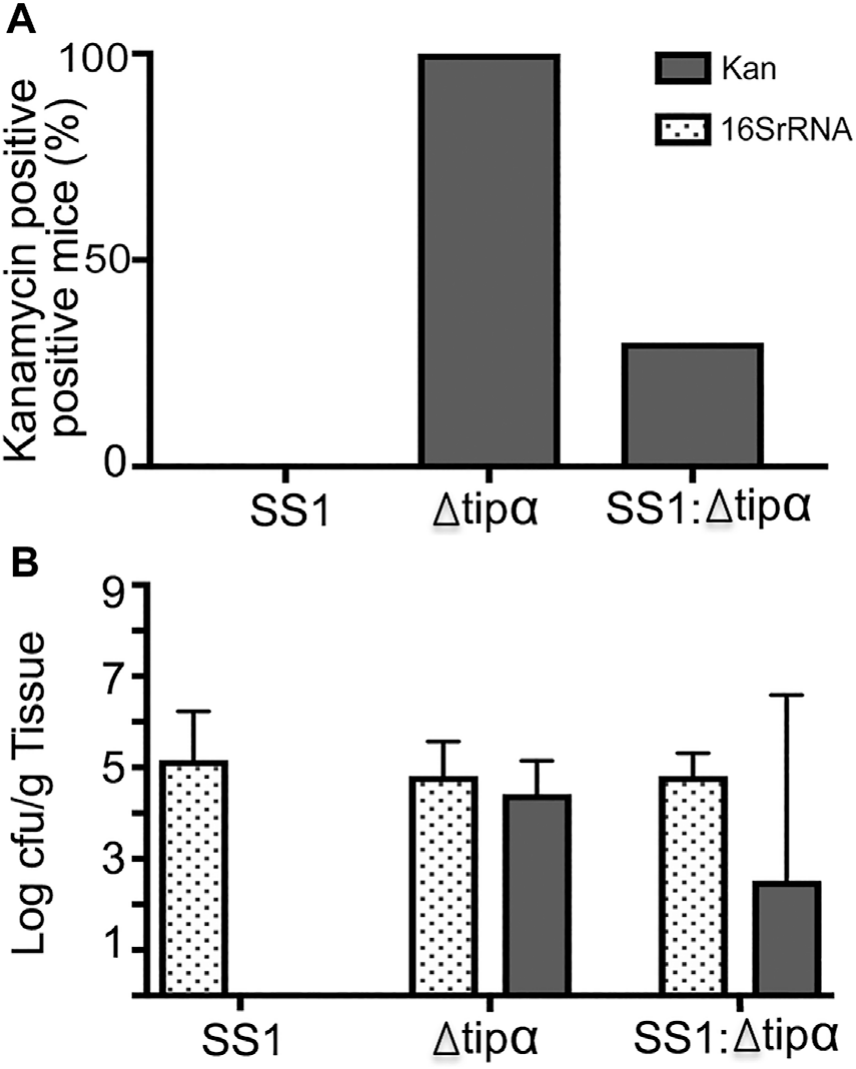
Quantifying ratio of WT SS1 to Δ*tipα* in co-infected mice at 4 months post infection (second infection). Kanamycin and *H. pylori* specific 16SrRNA primers were used for qPCR to quantify the amount of Δ*tipα* and the total bacterial load, respectively. **(A)** The percentage of Δ*tipα* positive mice in each group using kanamycin primers. **(B)** Total microbial load in each infected group determined by 16SrRNA primers with microbial load determination of the Δ*tipα* strain using kanamycin primers on a log scale.

The microbial load data demonstrates that knocking out Tipα expression does not inhibit the *Δtipα* strain from colonizing mice (Figure 10). It is possible however that if Tipα confers a growth or colonization benefit, deletion of Tipα could be a disadvantage in colonizing the gastric mucosa if competing with Tipα expressing *H. pylori*. Screening DNA purified from gastric tissue by PCR specific for the kanamycin resistance gene insert used to prevent Tipα expression, resulted in only three of the 10 co-infected mice testing positive (Figure 10A). Additionally, in those mice testing positive for the kanamycin resistance gene, the mutant strain was present at a reduced quantity in comparison to WT SS1 (Figure 10B). Specifically, for every 1 cfu of *Δtipα* per Gram of tissue, there were 100 cfu of WT SS1. The predominance of the WT SS1 strain in this group can explain the comparable cytokine mRNA expression and histology observed between the WT SS1 infected group and co-infected group.

## Discussion

Previous studies indicate that Tipα plays a large role in *H. pylori* induced inflammation and carcinogenesis. This is largely attributed to the induction of TNFα via NFκB (Suganuma et al., 2001; Cheng et al., 2008) and the increased expression of additional pro-inflammatory chemokines (Kuzuhara et al., 2007a). Tipα has also been linked to the epithelial- mesenchymal transition (EMT) promoting carcinogenesis in the MKN-1 gastric cancer cell line (Watanabe et al., 2014). The present experiments were performed to expand our understanding of how Tipα influences the host response using a mouse model of *H. pylori* infection. We demonstrate that Tipα contributes to *H. pylori*-induced inflammation and may play an important role in promoting gastric hyperplasia. The Δtipɑ-infected mice had significantly reduced expression of pro-inflammatory cytokines such as IFNγ, TNFα and KC based on mRNA levels. Because Tipα *in vitro* contributes to the induction of pro-inflammatory cytokines, we expected to see a reduction of these cytokines leading to reduced inflammation in the Δtipɑ infected mice. IFNγ and TNFα are secreted by Th1 cells and lower transcription levels in our mutant infected mice suggest that Tipα may play a role in inducing Th1 mediated immunity as well as acute inflammatory mechanisms.

The role of Tipα in promoting Th17 cell activity is less clear. Mice infected with WT *H. pylori* induced significantly more IL-17 than the Tipα-deficient in only one of our experiments (Figure 4). Both groups induced statistically comparable levels of IL-17 mRNA in the experiment shown in Figure 8, although in relative terms the Δtipɑ-infected mice expressed the highest. Heightened levels of IL-17 have been seen in *H. pylori* infected gastric mucosa (Luzza et al., 2000; Algood et al., 2007; Caruso et al., 2008; Shi et al., 2010), implicating the role of Th17 cells in the development of *H. pylori* induced gastritis (Shi et al., 2010). Consistent with these findings, our model shows mice infected with WT *H. pylori* develop a mixed Th1/Th17 immune response marked by the elevated levels of IFNγ and IL-17. Co-infected mice produced cytokine mRNA levels comparable to the WT infected mice.

Our molecular analysis on Tipα-stimulated mouse gastric epithelial cells is consistent with what has been described about *H. pylori* immunopathogenesis in general and Tipα activity specifically. IL-1, TNFα, IL-6 and IFNγ were all upregulated and have been well known to be involved in *H. pylori*-induced gastric inflammation. The acute phase response pathway and NFκB signaling pathways were also indicated. Although relatively few studies have ben performed on Tipα and its activity, it is known to bind cell surface nucleolin and induce the production of TNFα and other pro-inflammatory cytokines through NF-κB (Suganuma et al., 2001; Suganuma et al., 2005; Suganuma et al., 2006). Mahant et al. have used computer modeling to develop a model in which Tipα binding and internalization with nucleolin activates the Ras pathway leading to downstream NF-κB activity promoting inflammation. (Mahant et al., 2021). However, their model supports a role for Tipα monomers to interact with nucleolin in its inactive state which leads to proliferation, which creates an environment that supports hyperplasia.

The presence of Tipα was also associated with gastric hyperplasia. Foveolar hyperplasia is caused by chronic gastritis and is common during *H. pylori* infection. Although not a marker for cancer it does indicate the presence of cellular changes that can predispose an individual to gastric adenocarcinoma (Correa and Piazuelo, 2012). Our studies were performed using the Sydney Strain (SS1), one of the first well characterized mouse-adapted strains of *H. pylori* (Lee et al., 1997). Although a more recent isolate, Sydney Strain 2000, has been developed and put into widespread use due to its ability to induce increased inflammation (Thompson et al., 2004), we have employed the use of Hp SS1 for several reasons. First, the SS2000 lacks the entire cag pathogenicity island, an important virulence factor in *H. pylori* pathogenesis. Second, a comparison of the two strains in C57BL/6 mice demonstrate that the degree of atrophy, including mucosal hyperplasia, was equivalent (Thompson et al., 2004). Our Δtipɑ infected mice failed to develop hyperplasia after 4 months infection whereas some degree of hyperplasia was seen in both our WT and co-infected groups of mice. We demonstrate increased IL-1β involvement in our Tipα stimulated gastric cancer cells model. Overexpression of IL-1β in the gastric antrum of genetically modified mice has been demonstrated to be sufficient to suppress gastrin and induce gastric hyperplasia (Ding et al., 2021). The host response to *H. pylori* is complex. It involves multiple virulence factors, a regulated immune response concurrent to acute inflammatory events, length of infection and other factors. It is difficult therefore to ascertain the role of a single factor such as Tipα on this process. However, based on previous reports using *in vitro* analysis indicating Tipα is a pro-inflammatory factor, and our new mouse infection model in which lack Tipα resulted in loss of hyperplasia, we believe that Tipα contributes to the development of hyperplasia due to its ability to help promote inflammation.

Tipα may also provide a competitive advantage in colonization. In a previous 3 weeks *in vivo* study, an isogenic Tipα *H. pylori* mutant infected mice with a reduced bacterial load compared to mice infected with the parent WT SS1 strain (Godlewska et al., 2008). In our initial mouse infection, Tipɑ-deficient *H. pylori* infected mice with reduced loads in comparison to wild-type *H. pylori* at 1 month and 4 months of infection. We did not observe this difference between strains in our second experiment. However, our dual challenge model resulted in only three of 10 mice being infected by the Tipα-deficient bacteria whereas all 10 mice were infected with WT SS1. Additionally, in the mice in which the *Δtipα* strain did colonize, the WT bacteria were 100-fold more plentiful than the *Δtipα* strain. Although Tipα is a secreted virulence factor, the Tipα produced by the WT strain it did not seem to benefit the Tipα deficient strain. Given what little is currently known about Tipα, it is difficult to speculate as to why a deficiency in Tipα would be a disadvantage for colonization. The *Δtipα* strain induced less inflammation which, counterintuitively, may be interpreted to mean that increased inflammation may favor increased bacterial loads. However, previous reports using either *H. felis* or *H. pylori* have demonstrated that immunodeficient SCID mice had increased colonization compared to WT mice (Blanchard et al., 1995; Eaton et al., 1999; Nedrud et al., 2012). Additionally, no difference in bacterial load has been observed in HIV positive and negative patients despite the significance differences in host immune response (Spurnic et al., 2021).

Our *in vitro* analysis revealed genes associated with Tipα protein in mouse gastric epithelial cells. The potential contamination of purified recombinant proteins with *E.coli* LPS is known to complicate bioactivity analysis (Gao and Tsan, 2003; Cardoso et al., 2007), particularly when evaluating pro-inflammatory pathways. We were unable to eliminate LPS from our rTipα and therefore used Polymixin B (PMB) to neutralize the LPS (Palmer and Rifkind, 1974; Cardoso et al., 2007). The use of PMB greatly reduced the expression of TNFɑ and KC. Despite the decrease in expression of those genes, expression was still greater compared to unstimulated cells and we were able to identify 26 genes preferentially induced by rTipα, including TNFɑ, which has been described previously (Kuzuhara et al., 2007a; Cheng et al., 2008). Several of these genes are notable since many are nuclear proteins and transcription factors. Such proteins may represent specific targets since the Tipα protein has been demonstrated to enter the nucleus (Suganuma et al., 2008). Some nuclear. DNA binding assays have shown that Tipɑ is able to bind indiscriminately to DNA/RNA *in vitro* (Kuzuhara et al., 2007b). Therefore, proteins such as the transcription factor NR4A2 whose suppression has been linked to the gastrointestinal inflammation and adenocarcinoma (Han and Cao, 2012; Mohan et al., 2012; Yeh et al., 2016), and the A20 protein, an NFκB inhibitor (Kuzuhara et al., 2007a) may indicate promising candidates for further analysis on Tipα activity and pathogenesis.

These data are the first *in vivo* analyses demonstrating Tipα contributes to *H. pylori* associated gastric inflammation and are consistent with previous reports using *in vitro* models to demonstrate Tipα induces proinflammatory cytokine expression in epithelial cells (Suganuma et al., 2001; Suganuma et al., 2005; Kuzuhara et al., 2007a). These data also suggest that Tipα may contribute to hyperplasia. Additional studies are required to elucidate the long-term effects of Tipα expression in *H. pylori* infection on gastric pathology and disease progression and the unique mechanisms associated with this virulence factor.

## Summary

*Helicobacter pylori* (*H. pylori*) bacterium live in the lining of the stomach in approximately half of the world’s population. All infected individuals develop microscopic inflammation in the stomach tissue which persists for life unless treated. More than 80% of individuals do not develop any symptoms but continued infection is a risk factor for gastroduodenal diseases such as gastritis, gastric adenocarcinoma, peptic ulcer disease and MALT lymphoma. *H*. *pylori* penetrates the mucus layer to live on the surface of the cells that line the stomach. *H. pylori* produces proteins that induce inflammation including Tipα. Previous reports indicate Tipα induces proinflammatory host factors in cell culture that contribute to disease including carcinogenesis. We developed *H. pylori* with no Tipα and used a mouse model to investigate the role of Tipα in pathogenesis. We demonstrate that Tipα contributes significantly to the pro-inflammatory host response and the development of gastritis and hyperplasia, precursors to gastric adenocarcinoma. Genetic analysis of cell cultures treated with purified Tipα protein were consistent with these results. This report provides *in vivo* evidence that Tipα contributes to *H. pylori* pathogenesis and may play a role to promoting precancerous events that put the host at risk for developing gastric cancer.

## Data Availability

The original contributions presented in the study are included in the article/Supplementary Material, further inquiries can be directed to the corresponding author.

## Supplementary Material

Supplementary Figure S1
*Helicobacter pylori* recombinant Tipɑ (rTipα*)* protein expression from *E. coli.* Anti- 6X histidine antibody was used for the detection of rTipɑ in a western blot to confirm recombinant protein production. The 39 kDa band represents the Tipɑ homodimer and the 19 kDa band represents the monomeric form.

Supplementary Figure S2 Representative gastric histologic inflammation in H&E sections induced by wild type and *Δtipα H. pylori*. Top row: Representative images from the antral tissue of naïve, SS1-infected, and *Δtipα SS1 H. pylori* as indicated, demonstrating thickened mucosa and pronounced inflammatory infiltrate only in SS1 infected mice. Bottom row: Representative H&E-stained corpus tissue from each group demonstrating thickened mucosa in SS1 infected mice and inflammatory infiltrate. Magnification ×200.

Supplementary Figure S3 Representative images showing inflammatory infiltrate types in H&E sections Left: Chronic inflammation, characterized by mononuclear leukocytes displaying mainly round or ovoid nuclei. Right: Acute inflammation, characterized by polymorphonuclear leukocytes displaying multilobulated nuclei. (H&E, 600x).
