# Supplementary figures and images for: The TNF-Alpha Inducing Protein is Associated With Gastric Inflammation and Hyperplasia in a Murine Model of Helicobacter pylori Infection
Source: Front Pharmacol. 2022 Feb 14;13:817237. doi: 10.3389/fphar.2022.817237 (PMC8883333; doi:10.3389/fphar.2022.817237)

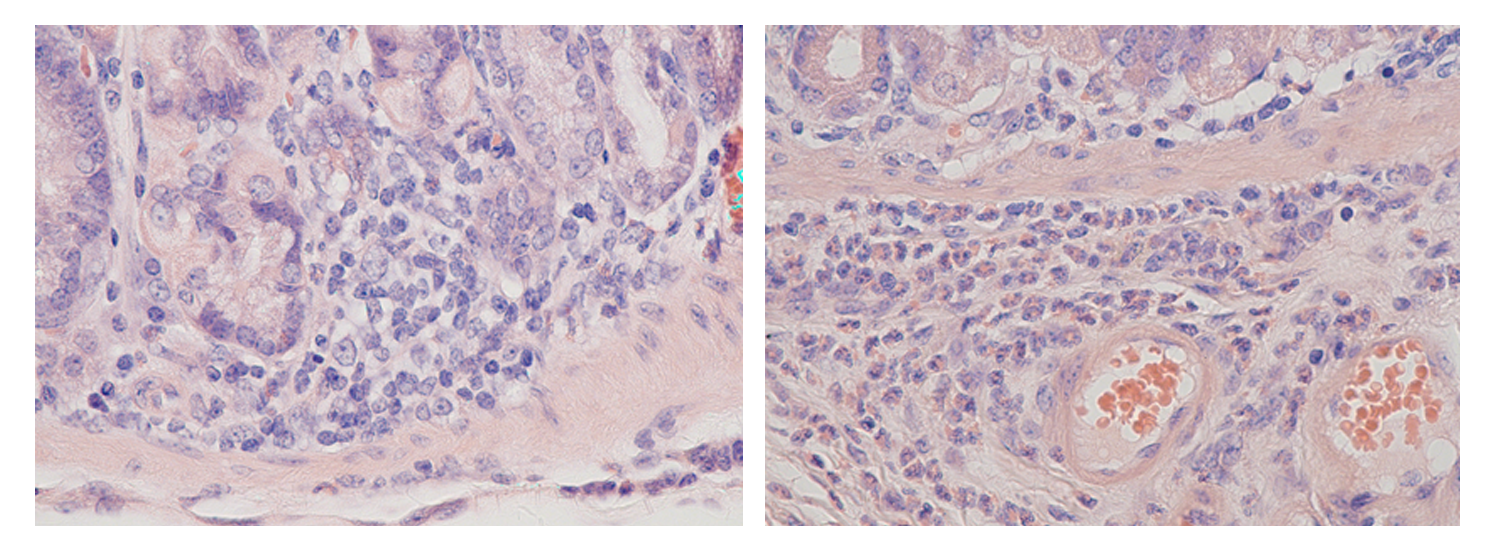

Supplement: Supplementary file 1 [file Image3.TIF]

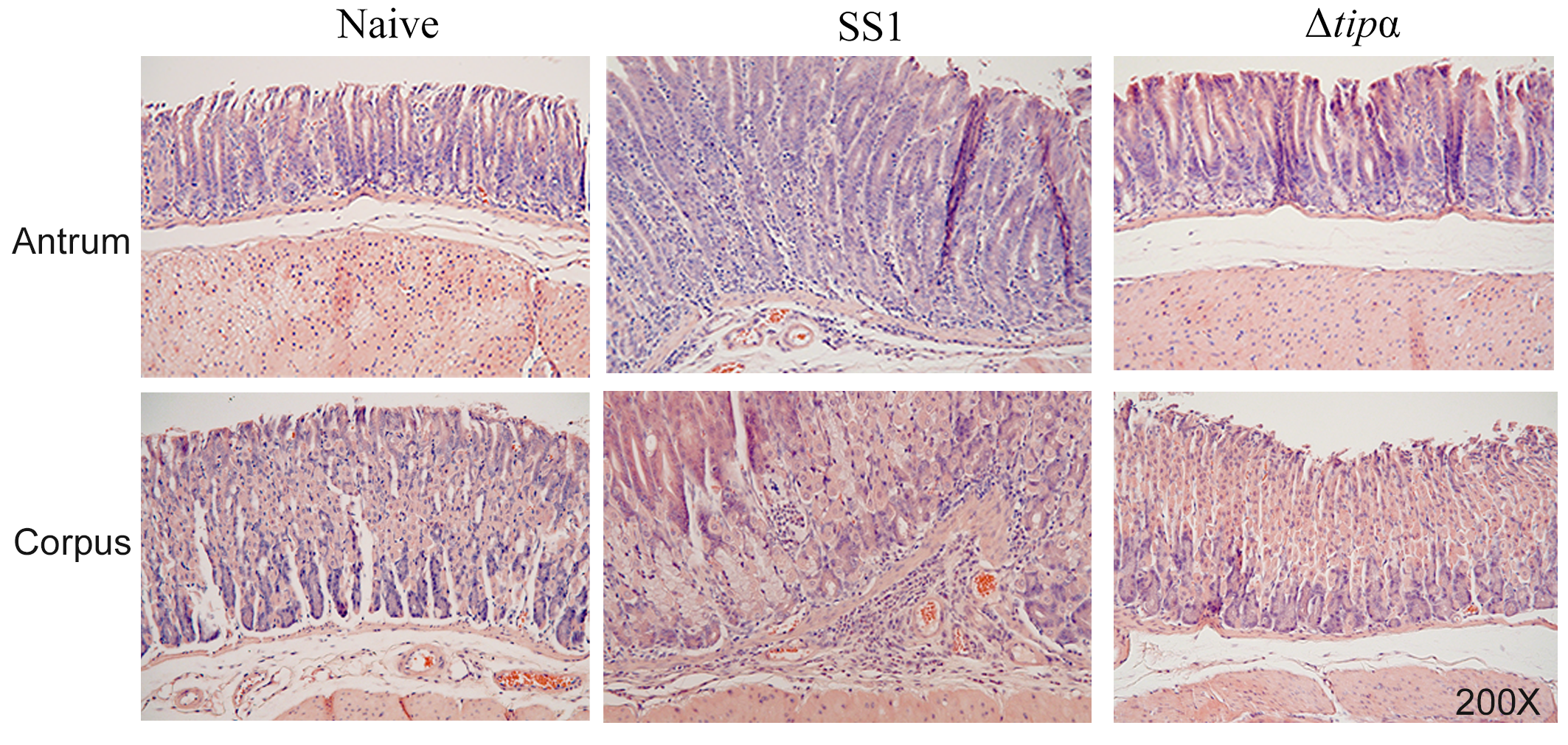

Supplement: Supplementary file 2 [file Image2.TIF]

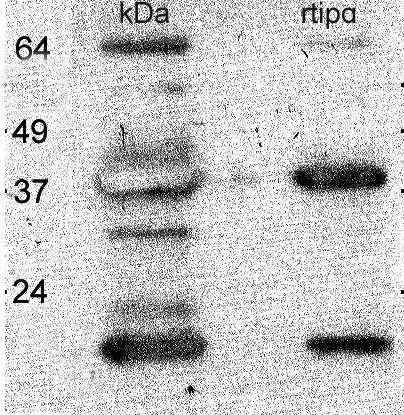

Supplement: Supplementary file 3 [file Image1.TIF]
